# The AIDS and Cancer Specimen Resource (ACSR): HIV malignancy specimens and data available at no cost

**DOI:** 10.1186/s12981-023-00558-4

**Published:** 2023-08-28

**Authors:** Sylvia Silver, Monika Schmelz

**Affiliations:** 1 Department of Microbiology, Immunology and Tropical Medicine, George Washington University, Washington, DC USA; 2 Department of Pathology, University of Arizona, 1501 N. Campbell Ave, Tucson, AZ 85724 USA

**Keywords:** HIV-related malignancy, Non-HIV related malignancy, Specimens, Biorepository

## Abstract

The goal of the AIDS and Cancer Specimen Resource (ACSR) is to play a major role in the advancement of HIV/AIDS cancer-related research/treatment by providing richly annotated biospecimens and data to researchers at no cost. The ACSR acquires, stores, and equitably distributes these samples and associated clinical data to investigators conducting HIV/AIDS-related research, at no costs. Currently, it is the only biorepository of human biospecimens from people with HIV and cancer available to eligible researchers globally who are studying HIV associated malignancies.

This review describes the history and organizational structure of the ACSR, its types of specimens in its inventory, and the process of requesting specimens. In addition, the review provides an overview of research that was performed over the last 5 years with its support and gives a summary of important new findings acquired by this research into the development of cancers in people with HIV, including both Aids-related and non-Aids-related malignancies.

## Mission of the AIDS and cancer specimen resource (ACSR)

The vision of the AIDS and Cancer Specimen Resource (ACSR) is to support the advancement of HIV/AIDS cancer-related research/treatment by providing richly annotated biospecimens and data to researchers *at no cost*. These materials are suitable for a broad range of projects that use traditional and cutting-edge technologies and analyses to address novel hypotheses. The ACSR’s mission is to acquire, store, and equitably distribute these samples and associated clinical data to investigators conducting HIV/AIDS-related research. Although the focus is on HIV and cancer, the inventory reflects other reactive and infectious processes collected in the mid to late 90’s that have been useful as controls and for studies of the HIV reservoir. The ACSR is the sole biospecimen resource for the confluence of HIV and cancer with a physical inventory that spans both the pre- and post-HAART (antiretroviral therapies) eras of the pandemic.

The purpose of this review is to provide a description of the ACSR, its inventory of biospecimens, and a summary of research publications that acknowledge grants of specimens and support from the ACSR.

## Evolution of the ACSR

At the time of its original funding by the NIH National Cancer Institute (NCI) in 1994, the AIDS Malignancy Bank (AMB) consisted of five institutions to capture representation of the HIV epidemic across the United States. These founding institutions (University of California, San Francisco [UCSF], the University of California, Los Angeles [UCLA], George Washington University [GW], State University of New York [SUNY-Brooklyn] and the Ohio State University [OSU]) geared collections strictly to specimens representing the then-prominent AIDS defining cancers (ADCs).

However, within five years the AMB began collecting specimens not only representing the presenting malignancy but additional types of specimens hoping to support the dynamics of cancer within the setting of “HIV disease,”. To reflect these additions to its inventory, the AMB name changed in 1999 to the AIDS and Cancer Specimen Bank (ACSB).

In 2002, further expansion of the ACSB’s inventory occurred through a collaboration with the NIH-funded Women’s Interagency HIV Study (WIHS); participants screened for cervical cancer could donate an extra colposcopy biopsy along with a blood specimen to the ACSB. In addition, the ACSB became a technological resource by offering collections of specimens through Tissue Microarrays (TMA). Its name was changed to the AIDS and Cancer Specimen Resource (ACSR) to reflect its support of cancer research. In 2013, ACSR’s funding became a single, consolidated, NIH UM1 award with substantial involvement of the NCI Office of HIV and AIDS Malignancy (OHAM) in the scientific and programmatic aspects of the group. The Office of the Chairs (OC) was established with overall responsibility for the structure, productivity, scientific and technical integrity, and fiscal accountability of the ACSR. The governance of the ACSR resides in its Executive Committee (EC), which is responsible for policy, priority setting, authorization of specimen acquisition and distributions, allocation of resources and performance reviews.

The present ACSR is comprised of the Baylor College of Medicine (BCM), George Washington University (GW), University of Arizona, Tucson (UArizona), University of California San Francisco (UCSF), Stellenbosch University (SU) in South Africa, and University of Sao Paulo (Brazil). A Technical Core resides at Mayo Clinic, Scottsdale (Arizona). All specimens were collected and received in accordance with the Declaration of Helsinki and were approved by appropriate ethics committees; the U.S. institutions operate under a single reliant IRB (Institutional Review Board) agreement. The figure below shows the organizational governance of the ACSR.

**Fig. 1:**
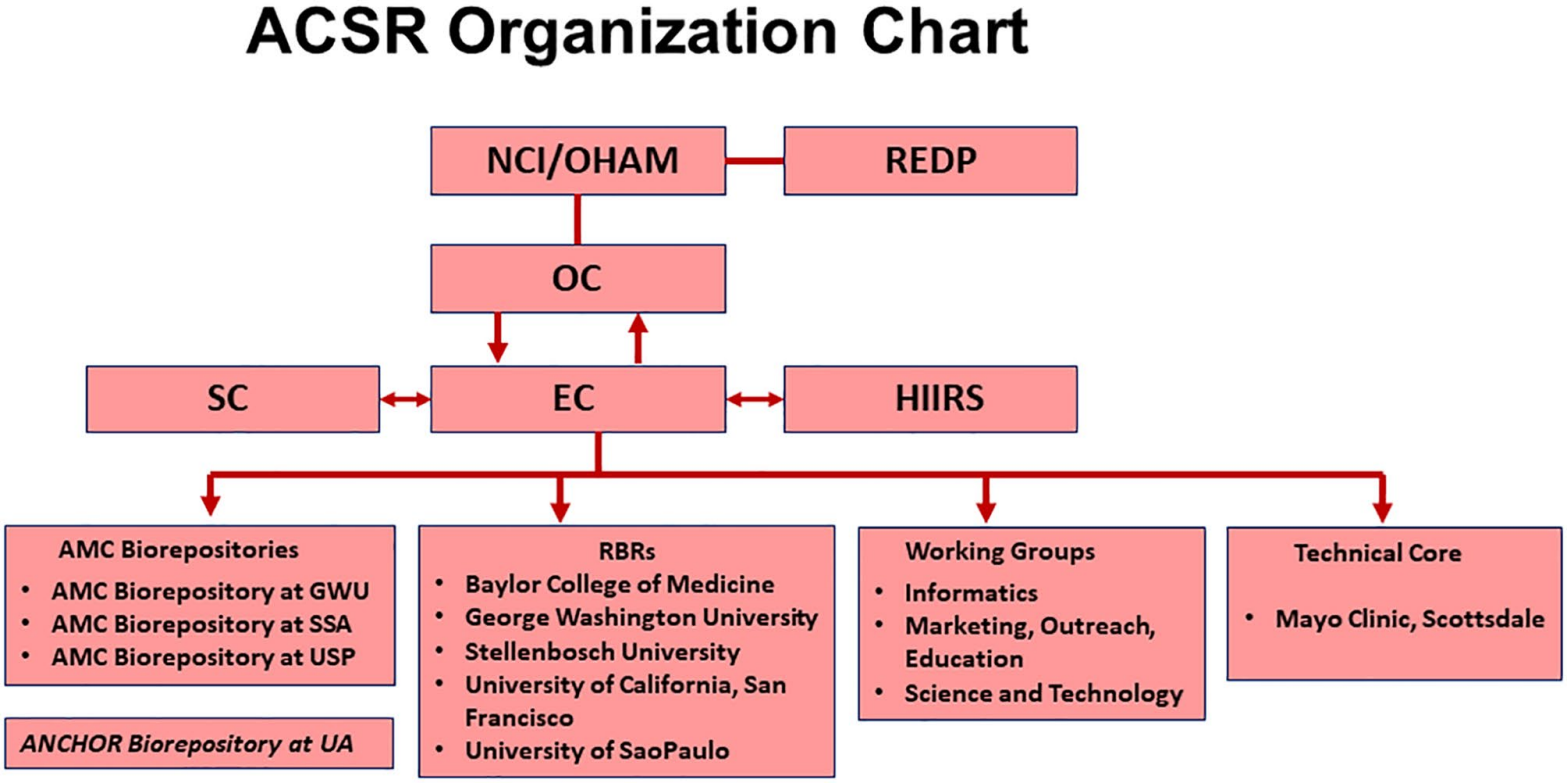
Organization Chart of the ACSR Abbreviations: **OHAM**-NCI/Office of HIV and AIDS Malignany; **REDP**-Review, Evaluation, and Decision Panel; **OC**-Office of the Chairs; **EC**- Executive Committee; **SC**-Steering Committee; **HIIRS**-Hub for integrated Informatics and Research Support; **AMC**-AIDS Malignancy Consortium; **SSA**- Sub-Saharan Africa; **ANCHOR**-Anal Cancer HSILOutcomes Research; **UA** University of Arizona; **RBRs**- Regional Biospecimen Repository; **GWU**- George Washington University; **USP**-University of Sao Paulo.

## Inventory characteristics of the ACSR

The ACSR includes biospecimens and annotated data from persons with and without HIV/AIDS as controls diagnosed with a wide spectrum of diseases and conditions, but particularly, malignancies. The current inventory includes blood components (serum, plasma, PBMCs, RNA, DNA) and tissues (fresh frozen, formalin fixed paraffin embedded blocks, slides, tissue microarrays). These specimen donations represent early- and late-stage disease with treatment histories that span the U.S. epidemic and two major international pandemic regions, namely, sub-Saharan Africa and Brazil in Latin America.

The ACSR’s main collaborator is the NCI/OHAM -supported, AIDS Malignancy Consortium (AMC). The latter performs phase I & II clinical trials into prevention, early detection, and treatment of HIV-related malignancies. Additional inventory includes clinical trial participant’s direct donations of blood, other body fluids and tissues originating from AMC clinical trials. AMC participants have the option of donating (through an IRB-approved consent protocol) at screening for an AMC trial or donation any of their left- over AMC specimens and clinical data generated from completed and closed studies.

Additionally, through national and international collaborations with the ACSR, specimens and data from several other large well-annotated collections are available, representing HIV and malignancies from the U.S. and sub-Saharan Africa. These other federally funded studies and their primary goals are as follows:

- *The Women’s Interagency HIV Study*: Multi-center, prospective, observational cohort study of cisgender women living with and without HIV in the United States.
- *San Francisco Young Men’s Health Study*: Prospective study of HIV infection in homosexual and bisexual men 18 to 29 years of age.
- *Antiretrovirals in Kaposi Sarcoma (ARKS)*: Study to compare treatments for HIV-related histologically-confirmed KS in adults in Uganda.
- *Uganda AIDS Rural Treatment Outcomes (UARTO)*: Prospective cohort study to describe the virologic, immunologic, and clinical outcomes of antiretroviral therapy and factors associated with these outcomes.
- *Epidemiology and Virology of KSHV in Zimbabwe*: Study to evaluate the prevalence and determinants of KSHV infection and the pattern of KSHV shedding.

## Research supported by the ACSR

The introduction of highly active combination antiretroviral therapy (HAART) significantly improved morbidity, mortality and the life expectancy of patients infected with HIV. Subsequently, while HAART dramatically improved the survival rate of patients living with HIV and AIDS, the numbers and age of this population has increased. Besides living with the consequences of HIV, increased age escalates the risk of cancer, thus making research in cancers associated with HIV/AIDS essential [1–4].

In the following sections, we provide summaries of selected published research outcomes supported by ACSR specimens and data. Although there is some interest in moving away from the terms “AIDS Defining Cancers (ADC) and NON-AIDS Defining Cancers (NADC)” we will use these classifications [5].

## AIDS defining cancers

AIDS-defining cancers include Kaposi sarcoma, non-Hodgkin lymphoma such as aggressive B-cell lymphoma and cervical cancer.

### Kaposi sarcoma and KSHV

Kaposi sarcoma (KS), a vascular tumor initiated by infection of endothelial cells (ECs) with KS-associated herpesvirus (KSHV), is dependent on sustained proinflammatory signals provided by intra-lesion leukocytes and continued infection of new ECs [6]. The sources of these cytokines and infectious virus within lesions are not fully understood. KS is enriched in mast cells. The incidence of Kaposi sarcoma (KS) in patients infected with HIV greatly decreased since the introduction of active antiretroviral therapy (ART) controlling HIV replication. However, there is growing evidence of a reemergence of Kaposi sarcoma in HIV-positive people causing the risk of development at 35-60-fold higher than in the general population [7]; this reemergence under successful HAART treatment raises questions about the heterogeneous and complex pathology of this disease [8]. Considering this context, the recent studies supported by the ACSR are significant for gaining new insights into pathogenesis and identifying new therapeutic targets.

#### Role of mast cells

Using in vitro and in vivo studies, Ayers et al. (2018) identified mast cells (MCs) as proinflammatory cells within KS lesions that are permissive for, and activated by, infection with KSHV, and as a potential long-lived reservoir for KSHV and a source of proinflammatory mediators within the KS lesion microenvironment [6]. Importantly, the study identifies MC antagonists as a promising novel therapeutic approach for KS [6].

Byakwaga et al. (2021) examined the activation of mast cells (MC) in a cross-sectional study of untreated PWH in a cohort in Uganda with or without KS [9]. The study found a dose-response relationship between plasma IgE levels and the presence and severity of KS, and that therapies targeting IgE-mediated MC activation might be a new treatment approach [9].

#### Viral circular RNAs in KS are unique biomarkers

A study by Toptan et al. (2018) showed that both the Epstein-Barr virus (EBV) and the Kaposi sarcoma herpes virus (KSHV) express circular RNAs (circRNAs) in tumors and cell lines infected with both viruses, which is a novel significant finding [10]. Until this study, it was not known whether human DNA viruses express circRNAs. Understanding the function of tumor virus circRNAs, which are unique biomarkers, may contribute to the knowledge about how these viruses cause cancer [10].

A study by Abere et al. (2020) showed that for one KSHV region, the PAN/K7.3 locus, broadly and bidirectionally generated circRNA levels that paralleled corresponding linear RNA levels, while another KSHV circRNA (circ-vIRF4) showed expression that differed from that of the corresponding linear RNA. The study also showed that all KSHV circRNAs are incorporated into KSHV virions and are potentially expressed as immediate early products in newly infected cells [11].

#### Identification of angio- and lympangiogenetic molecules as therapeutic targets

Thakker et al. (2018), for the first time, demonstrated epidermal growth factor-like domain (EGFL7) to be an important angiogenic molecule secreted and upregulated during KSHV infection that could be exploited for blocking KSHV associated malignancies in conjugation with other anti-angiogenic therapies [12].

Lee et al. (2018) showed that lymphangiogenic pathways are involved in KSHV infection and progression to KSHV-associated pathogenesis. This study reported for the first time that viral interferon regulatory factor 3 (vIRF3) is detected in over 40% of KS lesions and functioning as a proangiogenic factor, inducing hyper sprouting formation and abnormal growth of lymphatic endothelial cells in a histone deacetylase 5 (HDAC5) dependent manner, which is a signal-response regulator for vascular homeostasis [13].

#### Identification of biomarkers and other therapeutic targets

Cavallin et al. (2018) showed that KSHV lytic replication as well as the KSHV-oncogene vGPCR activates PDGFRA signaling through upregulation of its ligands PDGFA/B, and that blocking of PDGFRA signaling is anti-tumorigenic indicating that stable inhibition of PDFGR-signaling has potential for KS treatment [14].

A study by Kumar et al. (2019) demonstrated that KSHV infection induced the E3 ligase HACE1 protein to regulate KSHV-induced oxidative stress by promoting the activation of Nrf2 and nuclear translocation. Absence of HACE1 resulted in increased ROS, which facilitates virus entry, cellular death and reduced nuclear Nrf2, antioxidant, and viral gene expression. Taken together, these studies suggest that HACE1 can be a potential target to induce cell death of KSHV-infected cells [15].

Valiya Veettila et al. (2020) identified neuronal and neuroendocrine gene (NE) proteins characteristic of NE tumors that are upregulated in KSHV infected patient tissues, and which potentially may provide an avenue to escape host immune surveillance when expressed at immunologically privileged sites such as at the interface of neurons and endothelial cells. These newly identified NE gene products potentially could serve as biomarkers and therapeutic targets for KSHV infected cells [16].

#### Tumorigenesis

A study by Naipauer et al. (2020) revealed that CpG hypo-methylation of oncogenic and differentiation pathway predominantly promotes KSHV in vivo tumorigenesis, occurs and selects for pre-existing host mutations that allow the KSHV oncovirus to express oncogenic lytic genes by creating permissive environment for viral-induced innate immunity and inflammation, which provides a selective advantage in vivo conducive to tumorigenesis. The results of this study point out the mutagenic potential of KSHV indicating the existence of KSHV-induced oncogenic host mutations in KS lesions that could be selected upon treatment and impact AIDS-KS therapies [17].

Members of the ACSR, together with collaborators at the UPMC Hillman Cancer Center and School of Medicine in Pittsburgh reviewed the status of the bacteria-virus interactions in Kaposi’s sarcoma-associated herpesvirus (KSHV) infection and KSHV-driven cancers [18]. Due to immunosuppression, patients with KSHV are at an increased risk for bacterial infections. In addition, infection with distinct opportunistic bacterial species have been associated with increased cell proliferation and tumorigenesis in KSHV-induced cancers through activation of pro-survival and -mitogenic cell signaling pathways. Moreover, among patients coinfected by HIV and KSHV, patients with KS have distinct oral microbiota compared to non-KS patients elucidating the various mechanisms in which bacteria affect KSHV-associated pathogenesis, will help identify therapeutic targets for KSHV infection and KSHV-related cancers [18].

#### Non-Hodgkin lymphoma

A person with HIV is 10 to 20 times more likely to develop aggressive non-Hodgkin lymphoma (NHL) despite HAART and 5 to 26 times more likely to develop Hodgkin lymphoma, a non-AIDS defining neoplasm, than a person without HIV infection [19]. The clinical outcome in patients with HIV-NHL has improved, approaching that of the general population when standard-dose chemotherapy paradigms are used in conjunction with HAART [20, 21]. HIV-NHLs make up the majority of lymphoma diagnoses and represent a diverse set of malignancies. The most common HIV-NHL is diffuse large B cell lymphoma (DLBCL), which is 17-fold more likely to occur. Its clinical course is more aggressive and often presents at advanced stages in HIV infected patients as compared to HIV-negative patients. However, the molecular pathology driving the aggressive nature of DLBCL is still poorly understood. The following studies supported by specimens and data from the ACSR are shedding some light on AIDS-defined non-Hodgkin lymphoma and were made with the support of the ACSR.

#### HIV(+) GBC-DLBCL is a malignancy molecularly distinct from HIV(-) GBC-DLBCL

A retrospective study examined the transcriptional, genomic and protein expression differences between HIV(+) and HIV(-) germinal center B-cell (GCB) diffuse large B cell lymphoma (DLBCL) cases using digital gene expression analysis, array comparative genomic hybridization (CGH) and immunohistochemistry (IHC) [22]. The results show that genes associated with cell cycle progression, DNA replication and DNA damage repair are significantly upregulated in HIV(+) GCB-DLBCL compared to HIV(-) tumors. In contrast, genes associated with cell cycle inhibition and apoptosis regulating Bcl2 proteins were significantly decreased and have less copy number variations as HIV (-) tumors, as determined by array CGH data, indicating enhanced genomic stability in (HIV (+) tumors. In summary, this study shows that HIV(+) GBC-DLBCL is distinct from HIV(-) GBC-DLBCL in its molecular profile. In addition as compared to the HIV (-) tumor, the study revealed an overexpression of TRFC/CD71 mRNA, an iron uptake mediator and positive regulator of T- and B cell proliferation, in HIV (+) GCB DLBCL tumors resulting in a loss of both adaptive and innate immune signaling, as well as alterations in receptor signaling [22].

Using the Lymph2Cx diagnostic assay for Cell of Origin (COO) typing transcriptional differences between HIV (+) and HIV (-) GCB-DLBCL, Maguire et al. (2019) examined 40 cases [23]. Reduced BCL2, a negative prognostic DLBCL marker, was observed in HIV (+) DLBCL suggesting a reduced dependence on the pro-survival effects of BCL2 and a switch to a mechanism that prevents cycle inhibition and induction of apoptosis in HIV (+) GBC-DLBCL [23].

Further insights into lymphomagenesis in HIV positive people comes from a retrospective study of HIV (+) and HIV (-) DLBCL formalin-fixed paraffin-embedded patient tissues assessing expression of activation-induced cytidine deaminase (AID) levels, known to be upregulated in non-neoplastic B-cells in vitro, by Shponka et al. (2020) [24]. The study showed higher AID and DC-SIGN receptor expression levels in HIV (+) DLBCL compared to HIV (-) DLBCL suggesting involvement of both AID and potentially the DC-SIGN receptor-signaling pathway in HIV related pathogenesis of lymphoma [24].

#### Identification of therapeutic targets

Diffuse large B-cell lymphoma (DLBCL) is a heterogeneous disease, with a variable response to chemotherapy depending on, and not limited to, cell of origin, double/triple hit, or MYC/BCL-2 co-expression status. Similar to DLBCL, AIDS-related DLBCL (ARL) with non-germinal center histology or MYC expression reports poorer response to treatment. In the immunocompetent population with DLBCL, CD30 positivity defines a histology with improved survival, however, the characteristics and outcomes of ARL treated HIV (+) tumors expressing CD30 are not well studied.

Chaudry et al. ((2021) assessing 135 ARL patients showed that 30% expressed CD30 [25]. The CD30 positive cases were mostly a non-germinal center phenotype and had a strong correlation with EBV. No differences in survival were identified in this study, possibly due to the small numbers of patients assessed with survival data [25].

This is an important finding since CD30 is the target for Brentuximab vedotin (BV, ADCETRIS®), an antibody-drug conjugate (ADC) consisting of an anti-CD30 monoclonal antibody covalently linked to the microtubule-disrupting agent monomethyl auristatin E (MMAE) by a protease-cleavable linker [26–28]. Brentuximab vedotin is currently approved for classical Hodgkin lymphoma (CHL), anaplastic large cell lymphoma (ALCL) (another lymphoma where malignant cells uniformly express CD30), as well as CD30-expressing peripheral T-cell lymphomas and mycosis fungoides (MF) [ADCETRIS prescribing information]. The efficacy of Brentuximab vedotin (BV, ADCETRIS®) in ARL patients with DLBCL needs be examined in clinical trials.

Wong et al. (2019) found the kinase Tyro3 upregulated in different NHL subtypes and in primary effusion lymphoma (PEL) cell lines and exudates by using multiplexed inhibitor bead-mass spectrometry (MIB/MS) [29]. Tyro3 plays a pivotal role in cell survival in PEL, a viral lymphoma associated with Kaposi’s sarcoma-associated herpesvirus (KSHV). They also developed an inhibitor against Tyro3 named UNC3810A, which hindered cell growth in PEL, but not in other NHL subtypes where Tyro3 was not highly expressed. This indicates that Tyro3 is a potential therapeutic target [29].

#### T–Cell lymphoma

Although T cell NHLs are rare in people living with HIV, the existing data suggest a very poor prognosis with a median OS of 5–12 months [30]. HIV1 infection increases the risk of cancer, immunodeficiency, and co-infection with oncogenic viruses, such as EPS, KSHV and human papilloma virus, can cause clonal expansions of T cells in vivo. Mellors et al. (2021) showed that HIV-1 proviruses integrated in the first introns of signal transducer and activator of transcription 3 (STAT3) and lymphocyte-specific protein tyrosine kinase (LCK) can play a significant role in the development of T cell lymphomas [31]. The development of these cancers appears to be a multistep process involving additional nonviral mutations, which could help explain why T cell lymphomas are rare in persons with HIV-1 infection [31].

#### Cervical cancer

Cervical cancer is the most common cancer affecting sub-Saharan African women and is prevalent among HIV-positive (HIV+) individuals. No comprehensive profiling of cancer genomes, transcriptomes or epigenomes has been performed in this population thus far. Gagliardi et al. (2020) characterized 118 tumors from Ugandan patients, of whom 72 were HIV+, and performed extended mutation analysis on an additional 89 tumors [32]. The study detected human papillomavirus (HPV)-clade-specific differences in tumor DNA methylation, promoter- and enhancer-associated histone marks, gene expression and pathway dysregulation. Changes in histone modification at HPV integration events were found to be correlated with upregulation of nearby genes and endogenous retroviruses [32].

## Non-AIDS defining cancers

Non-AIDS-defining cancers (NADC) include Hodgkin lymphoma and cancers of the mouth, throat, liver, lung, and anus. In addition to HIV infection, other factors, such as older age, infection with other viruses (such as HPV or hepatitis B or C virus), and heavy alcohol or tobacco use, may increase the risk of developing a NADC.

While the occurrence of AIDS-defining cancer has remained relatively steady, the burden of non-AIDS-defining cancers (NADCs) has increased and is now becoming a rising cause of morbidity among people living with HIV (PLHIV) treated with antiretroviral therapy (HAART). A study analyzing trends since the year 2000 in France showed that cancer is the leading cause of death in HIV (+) people [33]. PLHIV have increased mortality for anal cancer, Hodgkin lymphoma, liver cancer, lung cancer and skin melanoma [34]. The ACSR supports research into understanding the mechanism of these NADC developments/progression and to find targets for urgently needed treatments.

## Biospecimen science support

### Exosomes

A study supported by the ACSR showed that exosomes released from HIV-infected T cells and those purified from blood of HIV-positive patients stimulate proliferation, migration, and invasion of oral/oropharyngeal and lung cancer cells [35]. The HIV transactivation response (TAR) element RNA in HIV-infected T-cell exosomes is responsible for promoting cancer cell proliferation and inducing expression of proto-oncogenes and Toll-like receptor 3 (TLR3)-inducible genes. The study further showed that HIV-infected T-cell exosomes rapidly enter recipient cells through epidermal growth factor receptor (EGFR) and stimulate ERK1/2 phosphorylation via the EGFR/TLR3 axis, indicating that TAR RNA-containing exosomes from HIV-infected T cells promote growth and progression of particular NADCs through activation of the ERK cascade in an EGFR/TLR3-dependent manner [35].

### Persistent virus reservoirs and a phylogenetic analysis of HIV sequence variation

Combined antiretroviral therapy (HAART) does inhibit HIV virus replication but does not eradicate it. It can persist for years and reestablish replication if treatment stops. These persistent tissue reservoirs of HIV prevent a cure, and it is important to identify and characterize the tissues involved in long term harboring of the HIV virus. Five studies supported by the ACSR provided some insight into HIV tissue reservoirs, and one study revealed new insights into phylogenetic sequence variations.

Nolan et al. (2022) by sequencing HIV *env-nef* in DNA and RNA isolated from plasma, peripheral blood mononuclear cells and tumor biopsies, before and after ART, found that HIV diversity and RNA expression in KS tumors can be maintained after HAART in the absence of detectable plasma viral loads as demonstrated in four sub-Saharan African patients [36]. Even after reducing plasma HIV viral load to undetectable levels and restored immune function after ART treatment, HIV in KS tumors continues to be transcriptionally and translationally active with the potential to positively influence tumor progression [36].

Another study supported by the ACSR, Nolan et al. (2018) used HIV *env-nef* single genome sequencing and HIV gag droplet digital PCR (ddPCR) to survey 50 tissues from five subjects on cART with no detectable plasma viral load at death [37]. The authors found that the spleen most consistently contained multiple pro-viral and expressed sequences (4/5 participants). Spleen-derived HIV demonstrated two distinct phylogenetic patterns: multiple identical sequences, often from different tissues, as well as diverse viral sequences on long terminal branches. The results suggested that the spleen, a lymphatic organ at the intersection of the immune and circulatory systems, might play a key role in viral persistence [37].

Asha et al. (2020) demonstrated that Lipoxin A4 (LXA4) secretion is strategically downregulated by KSHV in the host to establish latency [38]. LXA4 interacts with SMARCB1, a chromatin modulator and tumor suppressor protein controlling tumorigenic events associated with the hedgehog (hh) signaling pathway. Downregulation of LXA4 also reduces PD-L1 expression and thereby enables immune evasion of KSHV as a strategy to survive and persist in the host. This study also points to the therapeutic potential of LXA4 and its targetable receptor, AhR, in KSHV’s pathogenesis [38].

Vasquez et al. (2018) developed a new multiplexed in situ hybridization (ISH) (RNAscope) protocol to detect and quantify HIV-DNA (vDNA) and HIV-RNA (vRNA) in formalin-fixed paraffin-embedded (FFPE) human tissues in combination with immunofluorescence (IF) phenotyping of the infected cells and tissues. This approach is aimed at providing insights in the biology of HIV tissue reservoirs and potentially leading to a cure for HIV [39].

Lamers et al. (2019) contrasted modifications in the five variable domains (V1-V5) of the HIV envelope protein that are fundamental for cell entry [40]. A panel of 24 tissues from seven subjects with no measurable plasma viral load (NPVL) was compared to variable domains from 76 tissues from 15 subjects who had a detectable plasma viral load (PVL) at death. NPVL subject’s V1 and V2 domains were usually highly variable in length, whereas length variation in PVL sequences was more stable. Longer V1s contained more charged residues, whereas longer V2s were more glycosylated. Structural analysis demonstrated V1/V2 charge, and N-site additions/subtractions were localized to the CD4 binding pocket. This are significant findings since diversified envelopes in tissues during therapy may represent a mechanism for HIV persistence in tissues, as binding pocket complexity is associated with HIV that may escape neutralization, whereas shorter envelopes are associated with increased infectivity. Further analysis of tissue-derived envelope sequences may enable better understanding of potential immunological approaches targeting the persistent HIV reservoir [40].

A study by Moorad et al. (2022) contributes to the spotty knowledge of HIV virus sequence variation [41]. This study reports additional genomes from historical US patient samples and from African KS biopsies. It describes an assay that spans regions of the virus that cannot be covered by short read sequencing. These include the terminal repeats, the LANA repeats, and the origins of replication. A phylogenetic analysis, based on 107 genomes, identified three distinct clades; one containing isolates from USA/Europe/Japan collected in the 1990s and two of Sub-Saharan Africa isolates collected since 2010. This analysis indicates that the KSHV strains circulating today differ from the isolates collected at the height of the AIDS epidemic. This analysis helps experimental designs and potential vaccine studies [41].

## Treatment outcomes

### Research of effects of treatment in sub-saharan African cohorts

McCluskey et al. (2021) investigated the prevalence of pre-treatment integrase polymorphism, which is associated with resistance to integrase inhibitors (INSTIs) in an ART-naive cohort with diverse HIV-1 subtypes in ART pre-treatment plasma samples received from the Uganda AIDS Rural Treatment Outcomes (UARTO) cohort [42]. The researchers amplified HIV-1 integrase using nested-PCR and Sanger-sequenced (HXB2 4230–5093), and Stanford HIVdb v8.8 was used to infer clinically significant INSTI-associated mutations. Human leukocyte antigen (HLA) typing was performed for all study participants. No HIV-1 polymorphism associated with high levels of Dolutegravir (DTG) resistance, the first-line antiretroviral therapy), was detected in Uganda in the pre-DTG era, supporting the widespread implementation of DTG [42].

Two other studies examined the effect of ART therapy on systemic inflammation in a HIV (+) cohort in Uganda [43, 44]. In order to examine the association between ART adherence and inflammation in HIV(+) Ugandans, Castillo-Mancilla et al. (2018) examined concentrations of interleukin6 (IL-6), D-dimer, soluble sCD14, sCD163, kynurenine/tryptophan ratio and CD8 T-cell activation in the plasma of 282 participants in an Ugandan cohort at baseline with 6 months after ART treatment initiations for participants with undetectable plasma HIV RNA [43]. Higher ART adherence was associated with lower levels of biomarkers of inflammation, immune activation and coagulopathy among Ugandans living with HIV who achieved viral suppression shortly after HAART initiation. This suggests that HAART adherence could have biological consequences beyond viral suppression [43, 44].

## ACSR’s technical core

The ACSR thrives towards excellence. Its Technical Core at Mayo Clinic (Scottsdale, AZ) is at the forefront of assessing, standardizing, and establishing methods to ensure the best possible research outcome using ACSR specimens as discussed in two recent publications.

Molecular profiling of lymphoma samples has contributed enormously to our understanding of disease biology leading to detailed descriptions of diagnostic categories. These studies have also helped the field to recognize different subtypes of disease, different diseases that share similar cellular pathway perturbations, different immune responses, and different prognostic groups. While nearly all these discoveries were made using unfixed, snap-frozen materials, clinical biopsy materials are comprised of formalin-fixed and paraffin-embedded (FFPE) tissues. The study by Robetorye et al. (2019) describes the impact of molecular profiling on the field of lymphoma, the challenges associated with using FFPE tissues for downstream molecular diagnostic testing, the various molecular profiling techniques, and provides an example of the clinical application of a molecular profiling test of lymphoma using FFPE tissues [45].

Tissue microarrays (TMAs) are important tools to conserve precious tissue resources from increasingly smaller biopsies and to control experimental costs and variation across sample sets. The quality assurance assessment of TMA materials created at centralized biobanks has not been standardized. This study outlines two processes for the construction of tissue microarrays (“recipient block” and “tape” methods) and the associated pre-construction quality control measures (pathology review, protein and RNA assessment, map creation, and storage conditions) developed by the AIDS Cancer Specimen Resource (ACSR) Network’s Science and Technology Core. These steps provide a suggested framework for quality assessment that allows end-users, receiving materials from tissue banks, confidence in their experimental results [46].

## Conclusions

In the AIDS pandemic, the ACSR remains a major partner in HIV malignancy research by supporting both AIDS Defining Cancers (ADC) and non-AIDS -Defined Cancer (NADCs) research through the provision of specimens/data to approved researchers *at no cost*. Individuals requesting ACSR inventory must go through a peer-review process facilitated by the NCIOHAM and demonstrate Institutional review and adequate funding available to perform the proposed research.

The ACSR’s biospecimens encompasses a general collection of various types and numbers of samples donated by people living with or without HIV both nationally and internationally. Included are samples collected pre-and post HAART from patients with AIDS defining cancers, non-AIDS defining cancers, opportunistic infections, and reactive processes. The ACSR’s domestic collection includes specimens donated from the Women’s Interagency HIV Study (WIHS) and the San Francisco Young Men’s Health Study (SGYMHS). The ACSR’s African collections encompasses samples and data related to antiretrovirals In Kaposi Sarcoma (ARKS), Uganda AIDS Rural Treatment Outcomes (UARTO), Epidemiology and Virology of KSHV in Zimbabwe, and Transmission of KSHV to Children in South Africa. In addition, specimens from multiple site autopsy are available.

Tissue Microarrays (TMAs) can be requested and viewed online (https://www.acsr1.com/tissue-microarrays/). The TMAs come with a Quality Assessment Summary containing a pathologist’s review of the hematoxylin/eosin (H&E) stained slide to confirm presence or absence of tumor, and documentation of biological stains done to assess the tumor and nucleic acid integrity of the core. Instructions about how to request specimens can be found on the ACSR’s website (https://www.acsr1.com/request-specimens/).

The ACSR is truly a resource for HIV research.

## Data Availability

Not applicable.
